# A review of phytochemistry, metabolite changes, and medicinal uses of the common sunflower seed and sprouts (*Helianthus annuus* L.)

**DOI:** 10.1186/s13065-017-0328-7

**Published:** 2017-09-29

**Authors:** Shuangshuang Guo, Yan Ge, Kriskamol Na Jom

**Affiliations:** 1 Department of Food Science and Technology, Faculty of Agro-Industry, Kasetsart University, Bangkok, 10900 Thailand; 2 College of Economics and Management, Nanjing Agricultural University, Nanjing, 210035 China

**Keywords:** Sunflower seeds, Nutritive value, Chemical constituents, Metabolites, Biological activities

## Abstract

The sunflower (*Helianthus annuus* L.) seed and sprout is a ubiquitous crop with abundant nutrients and biological activities. This review summarizes the nutritional and medical importance currently recognized but under-researched concerning both seed and sprout highlighting the potential benefits of their phytochemical constituents including phenolic acids, flavonoids and tocopherols. Furthermore, the dynamic metabolite changes which occur during germination and biological activities are evaluated. The aim is to provide scientific evidence for improving the dietary and pharmaceutical applications of this common but popular crop as a functional food.

## Review

### Introduction

The common sunflower (*Helianthus annuus* L.) is a species of the Asteraceae family grown commercially worldwide offering a variety of nutritional and medicinal benefits. The sunflower seed, although used as a snack, salad garnish, and in some bakery goods, is primarily harvested for oil production, ranking in 4th position at world level (8% of 186 Mt oil in 2012) after palm (29%), soybean (22%) and oilseed rape (13%) [1]. The sunflower seed and sprout contain valuable antioxidant, antimicrobial, anti-inflammatory, antihypertensive, wound-healing, and cardiovascular benefits found in its phenolic compounds, flavonoids, polyunsaturated fatty acids, and vitamins [2]. It is used in ethnomedicine for treating a number of disease conditions including heart disease, bronchial, laryngeal and pulmonary infections, coughs and colds and in whooping cough [3]. These notable medicinal, nutritional, and culinary benefits have resulted in historical and growing popularity of the sunflower and its constituent parts worldwide.

Sunflower germination also produces important secondary compounds with potentially important roles in ecology, as well as the physiology, biosynthesis, and biodegradation of organisms. This review underscores the importance of increased research regarding the sunflower sprout, in particular, by summarizing the chemical constituents, dynamic changes, metabolite biological impact, and overall nutritional value of this common plant.

### Nutritional value of sunflower seed

The common sunflower seed, grown and consumed worldwide, supplies a multitude of nutritious components including protein, unsaturated fats, fiber, vitamins (especially E), selenium, copper, zinc, folate, iron, and more. It can be used as a cooking oil, enjoyed as a roasted or salted snack, dehulled and included as a confectionary nut, and because the sunflower seed is high in sulphuric amino acids, its meal is widely used as both livestock and pet feed [4].

Sunflower seeds are composed of approximately 20% protein, seed storage proteins provide the sulfur and nitrogen needed for seedling development after germination [5]. These sulfur-rich proteins are ideal for many human metabiological needs, including muscular and skeletal cell development, insulin production, and as an antioxidant. There are two main types of storage proteins in the sunflower seed, including 11S globulins and napin-type 2S albumins, 60% of which is water-soluble 2S albumins and the remainder being 11S globulins [6]. Various albumins have been reported to possess bactericidal [7] and fungicidal properties [8, 9]. The sunflower seed is also a valuable source for glutamine/glutamic acid, asparagine/aspartic acid, arginine, and cysteine, and is protein-rich with both a well-balanced amino acid content and low anti-nutritional properties [10]. The content of glutamic acid, aspartic acid and arginine is 26.91, 10.50, 9.75 g/100 g protein in sunflower meal, respectively. In addition, essential amino acids i.e. phenylalanine and tyrosine, leucine, methionine and cysteine, the amounts of which are 8.56, 6.18, 3.47 g/100 g protein [11]. Sunflower seeds when combined with wheat-based breads also significantly increase the quantity and quality of protein in bread [12].

Sunflower seed contains 35–42% oil and is naturally rich in linoleic acid (55–70%) and consequently poor in oleic acid (20–25%). [13]. Research shows that sunflower oil may reduce both total cholesterol and low-density lipoprotein (LDL) cholesterol and offer antioxidant properties [14]. Oleic acid is a monounsaturated omega-9 fatty acid capable of lowering triacylglycerides and low-density lipoprotein cholesterol levels, increasing high-density lipoprotein (HDL) cholesterol, and thereby lower the risk of heart attack. Oleic acid also shows a stronger relation with breast cancer. This strongest evidence comes from studies of southern European populations, in whom intake of oleic acid sources, appear to be protective [15]. Menendez et al. [16] further confirm that oleic acid could suppresses Her-2/neu (erbB-2) expression which is a gene involved in the development of breast cancer. Moreover, a high content of oleic acid increases the oil’s stability to oxidative degradation at high temperatures [17]. Hence, high oleic oil is used in the canned food industry [18] and as an additive lubricant for cars and textile industry equipment. One advantage of this high oleic acid sunflower oil is its higher degree of oxidative stability, which is desirable for frying purposes, refining and storage compared to oils low in oleic acid [19].

Sunflower seed is an especially rich source of polyunsaturated fatty acids (approximately 31.0%) compared to other oilseeds: safflower seed (28.2%), sesame (25.5%), flax (22.4%), cottonseed (18.1%), peanut (13.1%) and soy (3.5%) respectively [20]. Linoleic acid is an essential, polyunsaturated omega-6 fatty acid with 2 *cis* double bonds. Inverse association between omega-6 fatty acid intake and the risk of coronary heart disease has been proved [21]. Conjugated linoleic acid (CLA) is isomers of linoleic acid with conjugated double bonds [22], *cis*-9, *trans*-11-CLA (CLA1) and *trans*-10, *cis*-12-CLA (CLA2) are the most active isomers of conjugated linoleic acid, they exhibit several important physiological effects, including anticancer [23], antioxidant, anti-atherosclerosis [24], and anti-obesity [25] activities, as well as normalization of impaired glucose tolerance in animals and humans [26]. Today, biotechnological methods are a potential method to produce active isomers [27]. In order to produce CLA, Hosseini et al. [28] use sunflower oil and castor oil as cost-effective substrates, convert sunflower oil and castor oil to free fatty acids by using bacterial (*Lactobacillus plantarum*) lipase at different conditions. This method enables us to produce the highest concentration of CLA isomers with a mixture of two bioactive isomers including *cis*-9, *trans*-11- CLA (0.38 mg ml^−1^) and *trans*-10, *cis*-12-CLA (0.42 mg ml^−1^) from 8 mg ml^−1^ sunflower oil. From the aspect of nutrition, a diet rich in unsaturated fatty acids (both oleic and linoleic) has been recommended. It has been acknowledged that sunflower oil with high oleic acid content has positive nutritional qualities.

In addition to high oleic acid and linoleic acid content, the sunflower seed also contains significantly higher amounts of vitamin E (37.8 mg/100 g), compared to linseed, sesame seed, and soy (all of which contain less than 3 mg/100 g) and even peanut (10.1 mg/100 g) [29]. Vitamin E are considered as vital antioxidants, playing a role in preventing or controlling nonspecific reactions from various oxidizing species produced in normal metabolism.

### Chemical constituents

**Fig. 1:**
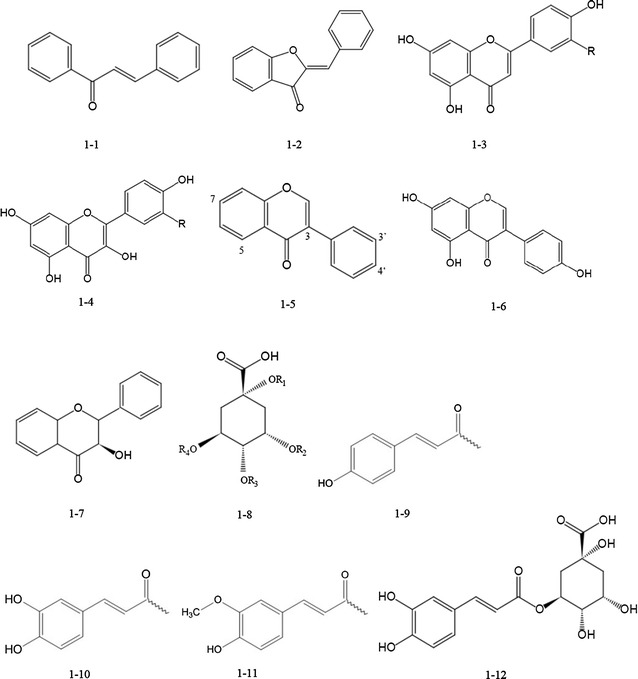
Structures of chemical components of Asteraceae. Chalcone [1-1], aurone [1-2], flavone: R=H apigenin, R=OH luteolin [1-3], flavonol: R=H kaempferol, R=OH quercetin [1-4], isoflavone [1-5], isoflavone (genistein) [1-6], dihydroflavonol [1-7], R_1_, R_2_, R3, R_4_=H: quinic acid [1-8], *p*-coumaroyl (pCo)[1-9], caffeoyl (C) [1-10], feruloyl (F) [1-11], 5-O-caffeoylquinic acid [1-12]

Edible seeds and sprouts are a good source of antioxidants, such as: flavonoids, phenolic acids, trace elements and vitamins [30]. During the past few decades, flavonoids (heliannone, quercetin, kaempferol, luteolin, apigenin) [31], phenolic acids (caffeic acid, chlorogenic acid, caffeoylquinic acid, gallic acid, protocatechuic, coumaric, ferulic acid, and sinapic acids) have been identified from the sunflower seed and sprout and have been shown to contribute to its pharmaceutical activities [32–34]. The structures of flavonoids and phenolic acids of Asteraceae are summarized in Fig. 1. Flavones and flavonols are the most commonly encountered flavonoid structural types in the Asteraceae family. The most widely occurring substitution patterns for flavones are 5,7,4′-trioxygenation (apigenin type) and 5,7,3′,4′-tetraoxygenation (luteolin type). For flavonols, 3,5,7,4′-tetraoxygenation (kaempferol type) and 3,5,7,3′,4′-pentaoxygenation (quercetion type) are most common [35].

### Flavonoids

Flavonoids are phenolic substances isolated from a wide range of vascular plants, which exhibit a wide range of biological benefits, including antibacterial, antiviral, anti-inflammatory, antiallergic, antithrombotic and vasodilatory [36]. The classes of flavonoids (flavanones, flavones, flavonols, isoflavonoids, anthocyanins, chalcone and aurone) vary in their structural characteristics around the heterocyclic oxygen ring. Flavonoids (Table 1) are the important metabolites found in the sunflower family. Among Japanese, flavonoid and isoflavone intake is the main component among nonnutrient phytochemicals with antioxidant potential in the diet. Aral et al. [37] demonstrate that a high consumption of both flavonoids and isoflavones by Japanese women may contribute to their low incidence of coronary heart disease compared with women in other countries. Isoflavone is a known phytoestrogen and has been reported to have various health beneficial roles such as antioxidation [38]. The total isoflavone content increases from 534 ng/g in the sunflower seed to 613.7 (soak in water) and 685.9 (soak in chitosan) ng/g after sprouting, which indicate that sunflower sprouts may offer a better functional food source than the raw sunflower seeds [39]. Flavonoid in the sunflower seed and sprout are 25 and 45 mg/g quercetin equivalent (the total flavonoids content in the extracts is compared to the standard curve for quercetin solutions and expressed as mg of quercetin equivalents per g dry matter of seeds and sprouts) [32]. The increase of total flavonoid contents in sunflower seeds during sprouting is in accordance with the results of Kim et al. [40]. These authors find that germination of mung bean causes the increase in flavonoid levels, compared to the intact seeds.

**Table 1:** Chemical constituents identified from sunflower family (Asteraceae)

Flavonoids	Skeleton	3	5	7	3′	4′	Refs
Kaempferol	[1-4]	OH	OH	OH	H	OH	[35]
Apigenin	[1-3]	H	OH	OH	H	OH	[35]
Dihydroflavonol	[1-7]	OH	H	H	H	H	[35]
Genistein	[1-6]	–	OH	OH	H	OH	[35]
Genistin	[1-5]	–	OH	Oglc	H	OH	[35]
Daidzein	[1-5]	–	H	OH	H	OH	[35]
Daidzin	[1-5]	–	H	Oglc	H	OH	[35]
Biochanin A	[1-5]	–	OH	OH	H	OCH_3_	[35]
Formononetin	[1-5]	–	H	OH	H	OCH_3_	[35]
Luteolin	[1-3]	H	OH	OH	OH	OH	[35]
Quercetin	[1-4]	OH	OH	OH	OH	OH	[35]
Phenolic acids		R1	R2	R3	R4		[33]
3-O-caffeoylquinic acid (3-CQA)	[1-8, 1-10]	H	C	H	H		[33]
5-O-caffeoylquinic acid (5-CQA)	[1-8, 1-10]	H	H	H	C		[33]
4-O-caffeoylquinic acid (4-CQA)	[1-8, 1-10]	H	H	C	H		[33]
5-O-p-coumaroylquinic acid	[1-8, 1-9]	H	H	H	*p*Co		[33]
5-O-feruloylquinic acid	[1-8, 1-11]	H	H	H	F		[33]
3,4-Di-o-caffeoylquinic acid (3,4-diCQA)	[1-8, 1-10]	H	C	C	H		[33]
3,5-Di-o-caffeoylquinic acid (3,5-diCQA)	[1-8, 1-10]	H	C	H	C		[33]
4,5-Di-o-caffeoylquinic acid (4,5-diCQA)	[1-8, 1-10]	H	H	C	C		[33]

Oglc, glucosyl; *p*Co, *p*-coumaroyl

### Phenolic acids

Phenolic acids occur in plants in different forms, such as aglycones (free phenolic acids), esters, glycosides, and/or bound complexes [41]. In Table 2, characteristic ions and contents of phenolic compounds identified in the sunflower seed are presented [33, 42]. It reports that 5-*O*-caffeoylquinic acid (5-CQA) is the predominant compound in non-oilseed and oilseed of sunflower, followed by diCQAs where gallic and ferulic acids are the predominant compounds in mung bean seed [43]. This CQA and its isomers 3- and 4-CQA, respectively, represent 62.1% up to 92.9% of the total phenolic content in all samples. The total phenolic content of the sunflower kernels of non-oilseed sunflowers is in a range of 3291.9–3611.0 mg/100 g DM, whereas oilseed kernels exhibites concentrations ranging from 3938.8 to 4175.9 mg/100 g DM [33]. Fisk et al. [44] find that total phenolic content is 2700 mg/100 g DM. Recent research shows that germination demonstratively influences the total, soluble, and bound phenolic contents in both seeds and especially sprouts [30]. Interestingly, germination increases total sunflower seed phenolic content by 232% [32], while research conducted by Cevallos-Casals and Cisneros-Zevallos [45] indicate a decrease in phenolic contents within the sunflower seed. These differences might be due to diversity among varieties, growing and storage conditions, and/or extraction procedures [40, 42]. Many studies indicate the high antioxidant potential of sunflower seed polyphenols (e.g. caffeic, chlorogenic, caffeoylyquinic, sinapic, ferulic, gallic, coumaric, and protocatechuic acids, glucoside, glucopyranoside, and cynarine) which remain when processed into an oil [32–34]. In contrast, phenolic compounds might reduce the quality of sunflower proteins by inhibiting digestibility, causing undesirable browning and structural modifications, and altering protein functional properties and behavior in various food matrixes.

**Table 2:** Characteristic ions and contents of phenolic acids of sunflower seed

Compounds name	Contents (mg/100 g of DM)		[M−H]^−^ (m/z)	Fragment ions (m/z)
	Non-oilseed	Oilseed		
Ferulic acid	7.6 ± 3.6	12.4 ± 2.0	193	193, 134
Caffeic acid	20.5 ± 1.6	26.7 ± 1.1	179	179, 135
Non-esterified phenolic acids	28.1 ± 4.0	39.0 ± 2.3		
3-*O*-caffeoylquinic acid	480 ± 21.6	439.9 ± 8.6	353	191, 179, 192,180, 135,134
4-*O*-caffeoylquinic acid	58.2 ± 0.8	87.5 ± 4.1	353	191, 179, 173, 135
5-*O*-caffeoylquinic acid	2795.7 ± 167.4	2467.0 ± 13.9	353	191, 179, 135
5-*O*-p-coumaroylquinic acid	11.3 ± 2.4	113 ± 1.0	337	191, 163
5-*O*-feruloyquinic acid	16.5 ± 1.5	113 ± 1.0	367	191, 173, 111, 193, 274, 336
Coumaric and ferulic acid derivative	27.9 ± 2.8	22.6 ± 1.4		
Dicaffeoylquinic acid	196.2 ± 7.0	360.9 ± 1.1	515	353, 335,191, 179, 173,135
Caffeoylquinic acid	24.7 ± 3.3	365 ± 22	353	191
Monocaffeoylquinic acids	3358.8 ± 168.8	3030.9 ± 17.0		
3,4-Di-*o*-caffeoylquinic acid	14.9 ± 5.8	28.8 ± 0.3	515	353, 173, 179, 498, 191, 354, 335, 203, 299
3,5-Di-*o*-caffeoylquinic acid	135.0 ± 3.0	211.2 ± 1.1	515	353, 191, 179, 135, 173
4,5-Di-*o*-caffeoylquinic acid	46.3 ± 2.7	120.9 ± 0.2	515	353, 173, 203, 179, 299, 255, 191, 335, 317

### Tocopherols

Vitamin E and other tocopherols are important sunflower oil components. Tocopherols are natural fat-soluble antioxidant vitamins viable both in vivo and in vitro [46]. There are four tocopherol derivatives: alpha, beta, gamma, and delta. These tocopherol isomers differ in their relative in vitro and in vivo antioxidant potency with alpha-tocopherol being highest. As an antioxidant, vitamin E performs various functions, possibly reducing the risk of cardiovascular disease and certain types of cancer [47]. Tocopherol, though essential for proper bodily function, cannot be synthesized in the human body, and therefore must be included in the diet [48].

Moderate amounts of tocopherols occur in cultivated sunflower seeds, predominantly alpha-tocopherol. Velasco et al. [49] in their research regarding commercial sunflower hybrids, report an average tocopherol content of 669.1 mg/kg, composed of alpha-tocopherol (92.4%), beta-tocopherol (5.6%), and gamma-tocopherol (2.0%). Nolascoa et al. [50] also report significant variations (389–1873 mg/g) in the total tocopherol concentration within sunflower seed oil depending on hull type, locations, hybrids, and radiation treatments. According to Fisk et al. [44], tocopherol values range from 214 to 392 mg/kg. In a more focused study, Rossi et al. [51] report alpha tocopherol content of 475 mg/100 g in the sunflower seed oil.

### Others

Sunflower seed and sprout contain high concentrations of niacin and vitamins A, B, and C. They are also rich in minerals, specifically calcium, iron, magnesium, phosphorus, potassium, selenium, and zinc [52] as well as cholesterol-lowering phytosterols. Notably, sprouts offer magnesium and zinc in much higher quantities than the seed. Luka et al. [53] report that sunflower seed extract revealed hypoglycaemic potential, possibly due to secondary metabolites, e.g. alkaloids, tannins, saponins, cardiac glycosides, terpenes, steroids and phenol.

### Dynamic changes in metabolites during sunflower seed sprouting

Macronutrient catabolism and degradation occurs during the sprouting process for carbohydrates, proteins, and lipids, accompanied by an increase of free amino acids and organic acids. Additionally, anti-nutritional and indigestible components, such as protease inhibitors and lectins, decrease during germination [54]. Finally, edible seeds experience an accumulation of some secondary metabolites, such as vitamin E and polyphenols.

Protease is responsible for converting proteins into amino acids [55] and the α-amylase enzyme converts starch into sugars. During germination, proteins and carbohydrates hydrolyze, with an accompanying increase of free amino acids and simple sugars. Erbas et al. [56] study two varieties of the sunflower seed and find that protein decreases from 48.1 and 40.9% to 35.5 and 28.4%, respectively, free amino acid content increases from 0.59 and 0.28% to 5.07 to 5.62% during sunflower seed. Total soluble and reducing sugar contents increase from 7.3 to 28.6 mg/g and 1.8 to 6.4 mg/g, respectively. Oil content increases during the initial stage of germination but decreases thereafter throughout seedling development with the most dramatic changes occurring between the 72 and 96 h mark. Free fatty acid content peaks at 72 h before decreasing. This may be due to an increase in oil hydrolysis, free fatty acid conversion to sucrose, and mobilization to the growing embryonic axis. The composition of the triglycerides also change, owing to their hydrolysis to free fatty acids originates and can be considered as a certain kind of pre-digestion [57].

Endogenous enzyme activation and complex biochemical metabolisms may lead to phenolic composition changes during germination. Several important molecular signaling pathways are involved in phenolic compound synthesis and transformation, including the oxidative pentose phosphate, acetate/malonate, phenylpropanoid, shikimate, hydrolysable tannin pathways, as well as glycolysis. Total phenolic content increases after 5 days of germination, the primary compounds being gallic, protocatechuic, caffeic, and sinapic acid along with quercetin. The quantities of the anti-nutritive components which affect the digestion of proteins reduce after germination, such as the flatulence-producing α-galactosides, trypsin and chymotrypsin inhibitors.

### Biological activities

The sunflower seed is a remarkable source of nutrients, minerals, antioxidants, and vitamins possessing antioxidant, antimicrobial, antidiabetic, antihypertensive, anti-inflammatory and wound-healing (Table 3). These various properties of this functional *H. annuus* L. are discussed below.

**Table 3:** Biological activities and compounds of sunflower seed and sprout

Biological activities	Biological compounds
Antioxidant effects	tocopherols, l-ascorbic acid, antioxidant enzymes catalase, glutathione dehydrogenase, guaiacol peroxidase, glutathione reductase, carotenoids
Antimicrobial activity	tannins, saponins, glycosides, alkaloids, phenolic compounds
Antidiabetic effects	chlorogenic acid, glycosides, phytosterols, caffeic acid, quinic acid
Antihypertensive effects	11S globulin peptides
Anti-inflammatory activity	α-tocopherol, triterpene glycosides, helianthosides
Wounds healing	linoleic acid, arachidonic acid

### Antioxidant effects

Antioxidants have long been recognized as having protective functions against cellular damage and reduce the risk of chronic diseases [58, 59]. Natural antioxidants occur as enzymes (catalase, glutathione dehydrogenase, and guaiacol peroxidase), peptides (reduced glutathione), carotenoids, and phenolic compounds (tocopherols, flavonoids and phenolic acids).

The antioxidant activity in the sunflower seedling is influenced by many factors. Antioxidant defenses may be affected by ultraviolet-B (UV-B) radiation absorbed in sunflower cotyledons. The soluble antioxidant defense (reduced glutathione) and antioxidant enzyme activities (catalase, glutathione dehydrogenase and guaiacol peroxidase) increase to 32.0 nmol/g, 0.36 pmol/mg, 4.6, and 18.7 U/mg in sunflower cotyledons exposed to 15 kJ/m^2^ UV-B, respectively [60]. Sunflower seeds exposed to saline demonstrated higher activities of antioxidant enzymes, including superoxide dismutase (SOD), guaiacol peroxidase (POD) and catalase (CAT) activity. Sunflower leaves in saline conditions exhibit higher activity of glutathione reductase (GR) and CAT activity than the root, while glutathione-S-transferase (GST), POD activity and SOD activity increased in the root compared to the leaf under the same conditions [61].

The antioxidant capacity of the striped sunflower seed cotyledon extracts has also been evaluated, the antioxidant capacity of ferric reducing/antioxidant power (FRAP), 2.2-diphenyl-1-picrylhydrazyl radical (DPPH) and oxygen radical absorbance capacity (ORAC) is 45.27 µmol; 50.18%, 1.5 Trolox equivalents, respectively [62]. During the sprouting phase, DPPH radical scavenging activity increases, probably due to the increased total phenolic, melatonin, and total isoflavone contents. The total phenolic content of the sunflower seed increases from 1.06 to 3.60 mg/g. Melatonin in the sunflower sprout is 1.44 ng/g, but is not detected in the seed. The total isoflavone content increases from 534 to 613.7 ng/g after germination [39]. Isoflavone has various health benefits as an antioxidant [38], an inhibitor for low-density lipoprotein (LDL) oxidation, and as a scavenger for DPPH radical activity [63]. Antioxidant activity of other seeds are generally found to increase during germination, the values of antioxidant activity increases almost 12-fold for mung bean, twice for radish, and by one-fifth of broccoli sprouts, when compared to the seeds [32].

### Antimicrobial activity

Nonspecific lipid transfer proteins (nsLTPs) belong to a large family of plant proteins. Lipid transfer protein (LTP) has strong antimicrobial activity against a model fungus. It is reported that LTP from onion is highly active against a broad range of fungi [64]. Ha-AP10 is a 10 kDa basic polypeptide homologous to many plant LTPs, which indicates effective antimicrobial activity against a model fungus. In the sunflower seed, as with other seeds, Ha-AP10 displayed high antifungal activity [65]. This protein is present during the first 5 days (and perhaps longer) of sunflower germination. Most of this is distributed in the cotyledons. Other report reveales that Ha-AP10 displays a weak inhibitory effect on *Alternaria alternata* fungus growth which naturally attacks the sunflower seed [66]. For these reasons, Ha-AP10’s role as an antifungal protein should be investigated further.

Parekh and Chanda [67] report that some secondary leaf and root metabolites inhibit certain microorganism growth isolated with sexually transmitted infections. Antimicrobial mechanisms vary between different phytochemicals. Tannins, for example, form irreversible complexes with proline-rich protein, resulting in the inhibition of microbial cell protein synthesis. Sunflower seed extract antibacterial and antifungal activity is studied by determining the inhibition zone formed around the disc revealing various degrees of potency for inhibiting *Salmonella typhi*, *Staphylococcus aureus*, *Bacillus subtilis*, *Vibrio cholera*, *Aspergillus fumigates*, *Rhizopus stolonifer*, *Candida albicans* and *Fusarium oxysporum* [68]. Antibacterial and antifungal activity may therefore be due to extracted flavonoids, alkaloids, saponins, and tannins which are proven to be inactivate microbial adhesions, enzymes, and cell envelope transport proteins [69]. The findings suggest that the extract from *H. annuus* seed has antimycobacterial activity (MIC = 500 μg/ml) [70] and this is agreed with a previous work by Cantrell et al. [71] who report that *I. helenium*, another specie in the sunflower family, has also the activity against *M. tuberculosis* H37Rv (100 μg/ml methanolic extract exceeds 80% inhibition using a radiorespirometric BACTEC assay).

### Antidiabetic effects

The formation and accumulation of advanced glycation end products (AGEs) under hyperglycemic conditions is a significant pathogenic contributor to diabetes [72]. Recently, substantial research is exploring the anti-AGE activities of natural foods. The sunflower sprout offers a diverse offense against AGEs. At 1.0 mg/mL concentration of extract, the AGE inhibitory rate of *H. annuus* L. is 83.29% [72]. Natural antioxidants and antiglycatives are more effective in treating and preventing diabetes [73], by eliminating the reactive oxygen species (ROS) which induce various biochemical pathways associated with diabetic complications. The sunflower sprout exhibits the most potent DPPH radical scavenging, iron-reducing, β-carotene oxidization inhibition compared to the seed. As a phenolic compound, cynarin possesses cholesterol/triglyceride-lowering effects and could potentially benefit patients with hyperglycemia or hyperlipidemia [74]. The cynarin content in the sunflower sprout is over 8% (w/w) which is much higher than that of artichoke leaves. Other phytochemicals, such as flavonoids, glycosides, and phytosterols are treats hypoglycaemic and anti-hyperglycaemic conditions [75].

The antidiabetic benefits of sunflower seed extract are studied in normal, glucose-loaded hyperglycemic- and streptozotocin- (STZ) induced type 2 diabetic rats. An extract dosage of 250 and 500 mg/kg reduce plasma glucose levels in normal rats 17.78 and 24.83% and 22.03 and 27.31% in diabetic rats, respectively. Luka et al. [53] also report that sunflower seed extract lowers plasma glucose levels. Sunflower seed extract (at two dosage 250 and 500 mg/kg) decrease blood glucose (*p* < 0.001) in streptozotocin-nicotinamide induced diabetic rats comparable to glibenclamide (600 μg/kg) while also improving body weight, liver glycogen content, glycosylated haemoglobin, plasma malondialdehyde, glutathione level, and serum insulin levels in diabetic rats [76]. Secondary metabolites in sunflower seed extract effectively controls glucose levels through alpha-glycosidase inhibitors which suppress intestinal brush border enzymes and thereby reduce carbohydrate digestion and absorption from the gut-postprandial hyperglycaemia [77].

### Antihypertensive effects

In recent years, bioactive peptides have been recognized as having biological advantages for digestion and observed during in vitro protein hydrolysis. Some bioactive peptides offer antihypertensive advantages by inhibiting the angio-tensin-I converting enzyme (ACE).

Sunflower protein hydrolysate is obtained through hydrolysis using pepsin and pancreatin. These peptides show different levels of ACE inhibitory effectiveness at different hydrolysis times. A significant increase in the generation of ACE inhibitory peptides occurs at the beginning of pepsin hydrolysis. Pancreatin hydrolysate also leads to maximum ACE inhibition in the beginning of hydrolysis [78]. Peptide is then purified and sequenced. After identifying the peptide by amino acid sequencing, it reveals a helianthinin fragment correspondence, namely the sunflower seed 11S globulin [79].

### Anti-inflammatory activity

Sunflower oil in anti-inflammatory and gastrointestinal profiles of indomethacin is evaluated in rats [80]. Results show that sunflower oil possesses significant anti-inflammatory benefits, possibly reducing carrageenan-induced paw edema by 79.5% compared to indomethacin (56.2%). Indomethacin is widely-used an anti-inflammatory drug, but the administration thereof causes notable gastric damage in rats. The administration of indomethacin together with sunflower oil causes no statistically significant gastric damage in rats. In fact, sunflower oil reduces oxidative damage in rat stomach tissues and therefore when combined with sunflower oil potentially prevents gastric damage. Other vegetable oils, such as olive oil, also offer anti-inflammatory effects via their constituents (tocopherols and steroids) [81, 82]. The presence of saponin in sunflower leaves reduces inflammation, as well.

### Wounds healing

Sunflower seed oil with a high concentration of linoleic acid can be indicated as a therapeutic alternative for both microscopical and clinical wound healing process in young male lambs [83]. After 3 days of the sunflower seed oil treatment, wound areas are reduced by 300% and after 7 days wounds improve macroscopically as well compared to control wounds [83]. These results confirm the efficiency of amino acids and essential fatty acids in wound healing reported by Baie and Sheikh [84]. Linoleic and arachidonic acids are not only important in the maintenance of a cutaneous barrier to water loss and as a precursor of prostaglandins, but also play a part in cell division regulation, epidermis differentiation, and consequently in the control of skin scaliness. Van Dorp [85] and Prottey et al. [86] observe that sunflower oil with a high linoleic acid content could reverse and cure both scaly lesions and dermatosis. Darmstadt et al. [87] test the impact of topical application of sunflower seed oil 3 times daily to preterm infants < 34 weeks’ gestational age on skin condition, treatments with sunflower seed oil result in a significant improvement in skin condition and a highly significant reduction in the incidence of nosocomial infections.

## Conclusions

The sunflower seed (*H. annuus* L.), though native to North America, is grown worldwide, being highly adaptable to climate, temperature, and light. Despite the sunflower seed and sprout’s growing demand and versatility in agriculture, diet, and even medicine, it remains under-researched with many untapped benefits to human health.

Germination not only alters the appearance, flavor, and taste of the seed, but, more importantly, amplifies its already valuable nutritional value [88]. The lipid, protein, and carbohydrate transformations, as well as the active compound syntheses which occur during this stage, provide ample areas for research, potentially leading to important human nutritional and pharmacological benefits. Therefore, addition research into this already high-demand food source is required to more fully understand and exploit the human health benefits of this versatile and economical crop as a functional food capable of treating a variety of ailments and dietary needs.

## Abbreviations

LDL: low-density lipoprotein
HDL: high-density lipoprotein
CLA: conjugated linoleic acid
CLA1: *cis*-9, *trans*-11-CLA
CLA2: *trans*-10, *cis*-12-CLA
Oglc: glucosyl
*p*Co: *p*-coumaroyl
CQA: caffeoylquinic acid
diCQA: di-o-caffeoylquinic acid
UV: ultraviolet
SOD: superoxide dismutase
POD: guaiacol peroxidase
CAT: catalase
GR: glutathione reductase
GST: glutathione-S-transferase
FRAP: ferric reducing/antioxidant power
ORAC: oxygen radical absorbance capacity
DPPH: l-diphenyl-2-picrylhydrazyl
nsLTPs: nonspecific lipid transfer proteins
LTP: lipid transfer protein
AGEs: advanced glycation end products
ROS: reactive oxygen species
STZ: streptozotocin
ACE: angio-tensin-I converting enzyme
